# Identifying the Signatures and Rules of Circulating Extracellular MicroRNA for Distinguishing Cancer Subtypes

**DOI:** 10.3389/fgene.2021.651610

**Published:** 2021-03-09

**Authors:** Fei Yuan, Zhandong Li, Lei Chen, Tao Zeng, Yu-Hang Zhang, Shijian Ding, Tao Huang, Yu-Dong Cai

**Affiliations:** ^1^School of Life Sciences, Shanghai University, Shanghai, China; ^2^Department of Science and Technology, Binzhou Medical University Hospital, Binzhou, China; ^3^College of Food Engineering, Jilin Engineering Normal University, Changchun, China; ^4^College of Information Engineering, Shanghai Maritime University, Shanghai, China; ^5^Bio-Med Big Data Center, CAS Key Laboratory of Computational Biology, Shanghai Institute of Nutrition and Health, University of Chinese Academy of Sciences, Chinese Academy of Sciences, Shanghai, China; ^6^Channing Division of Network Medicine, Brigham and Women’s Hospital, Harvard Medical School, Boston, MA, United States; ^7^CAS Key Laboratory of Tissue Microenvironment and Tumor, Shanghai Institute of Nutrition and Health, University of Chinese Academy of Sciences, Chinese Academy of Sciences, Shanghai, China

**Keywords:** circulating extracellular microRNA, signature, rule, cancer, subtype

## Abstract

Cancer is one of the most threatening diseases to humans. It can invade multiple significant organs, including lung, liver, stomach, pancreas, and even brain. The identification of cancer biomarkers is one of the most significant components of cancer studies as the foundation of clinical cancer diagnosis and related drug development. During the large-scale screening for cancer prevention and early diagnosis, obtaining cancer-related tissues is impossible. Thus, the identification of cancer-associated circulating biomarkers from liquid biopsy targeting has been proposed and has become the most important direction for research on clinical cancer diagnosis. Here, we analyzed pan-cancer extracellular microRNA profiles by using multiple machine-learning models. The extracellular microRNA profiles on 11 cancer types and non-cancer were first analyzed by Boruta to extract important microRNAs. Selected microRNAs were then evaluated by the Max-Relevance and Min-Redundancy feature selection method, resulting in a feature list, which were fed into the incremental feature selection method to identify candidate circulating extracellular microRNA for cancer recognition and classification. A series of quantitative classification rules was also established for such cancer classification, thereby providing a solid research foundation for further biomarker exploration and functional analyses of tumorigenesis at the level of circulating extracellular microRNA.

## Introduction

Cancer is one of the most threatening diseases to humans in the 21st century (Jemal et al., 2011; Siegel et al., 2019). Cancer is regarded as the second most deadly disease following cardiovascular diseases as it can invade multiple significant organs, including lung, liver, stomach, pancreas, and even brain. According to the World Health Organization’s statistics in 2018 (Bray et al., 2018), more than 18 million new cases and about 1 million deaths due to cancer exist globally. Accordingly, numerous studies have been conducted on the pathological mechanisms, clinical diagnosis, and treatment of cancer. Indeed, great achievements have been made in this field.

In particular, the identification of cancer biomarkers is regarded as one of the most significant parts of cancer studies as the foundation of clinical cancer diagnosis (Griffith et al., 2008; Ribaut et al., 2017) and related drug development (Jørgensen, 2019). Previously, researchers have revealed multiple cancer-subtype specific biomarkers by using genomics, transcriptomics, proteomics, or even multi-omic datasets (e.g., specific biomarkers of different cancer subtypes) at different biological omic levels. At the genomic level, specific biomarkers such as EGFR (Blakely et al., 2017) and KRAS (Arbour et al., 2018) exist for lung cancer, TP53 (Long et al., 2019) and LRP1B (Wang et al., 2019) for liver cancer, and BRAF (Ribas et al., 2019) and TP53 (Xiao et al., 2018) for skin melanoma. At the transcriptomic level, apart from the transcripts of already identified genomic biomarkers, multiple noncoding transcripts including microRNAs (e.g., hsa-miR-195-5p) (Li L. et al., 2020) and long non-coding RNAs (e.g., FOXE1 and HOXB13-AS1_2) for lung cancers have also been confirmed to be effective biomarkers for cancer diagnosis and classification (Li et al., 2019). With the development of biotechnology and biostatistics, cancer biomarkers at the proteomic level or even at the integrated multi-omic level have also been identified. For instance, in 2014, a systematic multi-omic analyses (Li et al., 2014) on lung cancer have revealed a group of potential multi-omic biomarkers for lung cancer, including EGFR and CCT6A. Analyzing data at different omics can improve accuracy and efficacy for potential biomarker identification. However, almost all such studies are based on cancer tissue *in situ*. In fact, during the large-scale screening for cancer prevention and early diagnosis, obtaining cancer-related tissues is impossible. To solve this problem, cancer-associated circulating biomarkers from liquid biopsy targeting have been presented, which has become one of the most important directions of clinical cancer diagnosis studies.

In the field of cancer-associated liquid biopsy, many research subdirections target biomarkers of different levels, such as cell-free DNA, plasma protein, and circulating RNAs. In particular, circulating RNAs have been extensively reported to be effective for cancer diagnosis or even classification. In 2004, researchers have shown that circulating plasma RNA may be a potential source of biomarkers for cancer screening (El-Hefnawy et al., 2004). In 2012, a systematic review has summarized the specificity and sensitivity of extracellular circulating RNAs to diagnosis and monitor different cancer subtypes (Zen and Zhang, 2012). In 2018, a study (Yokoi et al., 2018) integrating extracellular microRNA from serum for the diagnosis of ovarian cancer has demonstrated that extracellular microRNA biomarkers may distinguish one cancer subgroup from normal controls and contribute to the detailed cancer classification by comparing different cancer subgroups. These findings indicates that circulating extracellular microRNA may also be a specific “level/omics” of data that are sufficiently effective for cancer diagnosis and classification.

In the present study, based on shared data from a previous study (Yokoi et al., 2018), we performed an effective feature-selection procedure to identify candidate biomarkers for cancer recognition and classification by using multiple machine-learning models. The data was first analyzed by the Boruta (Kursa and Rudnicki, 2010) method to extract important microRNAs. Then, Max-Relevance and Min-Redundancy (mRMR) (Peng et al., 2005) feature selection method followed to evaluate the importance of each selected feature and ranked them in a feature list. Such list was fed into the incremental feature selection (IFS) (Liu and Setiono, 1998) method, incorporating one of the four classification algorithms, to extract latent microRNA biomarkers and build efficient classifiers. Additionally, a series of quantitative classification rules for cancer classification was established. This re-analysis on the extracellular microRNA dataset enabled the identification of a group of potential biomarkers for qualitative or quantitative cancer classification and laid a solid research foundation for further biomarker exploration and functional analyses of tumorigenesis at the circulating extracellular microRNA level.

## Materials and Methods

### Data

We downloaded the extracellular microRNA profiles of various cancers and non-cancer samples from Gene Expression Omnibus with accession number GSE106817^1^ (Yokoi et al., 2018); 4046 samples were included in such dataset and classified into 12 classes, including 11 cancer types and non-cancer class. The sample size of each class is given in Table 1. For each sample, the expression levels of 2565 microRNAs were measured with 3D-Gene Human miRNA V21_1.0.0. To accelerate the precision diagnosis of pan-cancer, we built a computational pipeline for extracellular microRNA-based cancer detection and classification.

**TABLE 1:** Statistic of samples used in this study.

Index	Class	Sample size
1	Benign ovarian disease	29
2	Borderline ovarian tumor	66
3	Breast cancer	115
4	Colorectal cancer	115
5	Esophageal cancer	88
6	Gastric cancer	115
7	Hepatocellular carcinoma	81
8	Lung cancer	115
9	Non-cancer	2759
10	Ovarian cancer	333
11	Pancreatic cancer	115
12	Sarcoma	115
In total		4046

### Boruta Feature Filtering

In the investigated dataset, lots of microRNAs (features) were involved. Evidently, not all microRNAs are related to the investigated cancer types. It is necessary to extract important ones and discard others. Here, we employed Boruta (Kursa and Rudnicki, 2010) method to quickly select relevant features with particular class labels (e.g., cancer types or non-cancer class). This method has been applied to deal with different biological and medical problems (Pan et al., 2020; Yuan et al., 2020; Zhang et al., 2021a).

Boruta is a random forest (RF)-based feature filtering method. Its computation steps included the following steps: (1) creation of shuffled data with shuffling original features in the original dataset, (2) evaluation of feature importance by comparing the RF on the original and shuffled data, (3) calculation of Z score for each feature depending on the feature’s importance score, (4) determination of the important feature by comparing its Z score with those of the shadow features, and (5) the above procedures stop until one of the following conditions was satisfied: (i) each feature is tagged as either “important” or “unimportant” and (ii) a predefined number of iterations is reached. The features tagged by “important” were kept for further analysis.

This study adopted the Boruta program obtained from https://github.com/scikit-learn-contrib/boruta_py, which was implemented by Python. For convenience, default parameters were used.

### Max-Relevance and Min-Redundancy Feature Selection

For the features selected by the Boruta method, mRMR (Peng et al., 2005) feature selection method was adopted to evaluate their importance. This method has wide applications in tackling several biological and medical problems (Chen et al., 2018, 2020; Zhao et al., 2018; Li M. et al., 2020; Pan et al., 2021).

mRMR method employed the Max-Relevance and Min-Redundancy to assess the importance of features. Features with high relevance to class labels and low redundancy to other features were termed to be important. To quantify the relevance and redundancy, it uses mutual information (MI). For two variables *x* and *y*, the MI score between them is defined by:

(1) $$I\,\left(x,y\right)=\iint p\,\left(x,y\right)\,\log\frac{p\,\left(x,y\right)}{p\,\left(x\right)\,p\,\left(y\right)}\,d\,x\,d\,y$$

where *p*(*x*)/*p*(*y*) and *p*(*x*,*y*) represent the marginal probabilistic density of *x/y* and joint probabilistic density of *x* and *y*, respectively. The mRMR method evaluates the importance of features by listing them in a feature list. A loop procedure is performed to produce the list. At first, this list is empty. For each feature not in the list, calculate its relevance to class labels, measured by the MI score of it and class label variable, and its redundancy to features in the list, measured by the average MI scores between it and features in the list. The feature with highest difference of relevance and redundancy is picked up and added to the list. When all features are in the list, the loop stops. This list was called mRMR feature list in this study. The combination of some top features can be the optimum feature space for a given classification algorithm.

The current study adopted the mRMR program retrieved from http://penglab.janelia.org/proj/mRMR/. Likewise, default parameters were used.

### Incremental Feature Selection

mRMR method only provided a feature list. It was still a problem for selecting optimum features for a given classification algorithm. Thus, we employed the IFS method (Liu and Setiono, 1998; Zhang et al., 2021b).

Using the mRMR feature list from the above mRMR, a series of feature subsets can be produced with a step interval as one. For example, the first feature subset includes the first feature in the list, and the second feature subset includes the first two features, and so on. Each classifier is then trained on the training data, in which samples are represented by features in one feature subset. Then, each classifier is evaluated by 10-fold cross-validation (Kohavi, 1995). The classifier with the best performance is selected and termed as the optimum classifier. The corresponding feature subset is determined as the optimal one.

### Synthetic Minority Oversampling Technique

Considering the used extracellular microRNA dataset has remarkably different numbers of samples (see Table 1), synthetic minority oversampling technique (SMOTE) (Chawla et al., 2002) was performed to produce sufficient new samples for minor classes. When evaluating the performance of a classifier with ten-fold cross-validation, we used SMOTE to create a new dataset with an equal sample number of different classes. For this analysis, the “SMOTE” tool in Weka software^2^ (Frank et al., 2004; Witten and Frank, 2005) was used.

### Classification Algorithm

To execute the IFS method, one classification algorithm is necessary. In this study, we tried four classification algorithms: RF (Breiman, 2001), support vector machine (SVM) (Cortes and Vapnik, 1995), k-nearest neighbor (kNN) (Cover and Hart, 1967), and decision tree (DT) (Safavian and Landgrebe, 1991). These algorithms have wide applications in tackling different problems (Ben-Hur et al., 2008; Ahmed et al., 2013; Chen et al., 2017; Sankari and Manimegalai, 2018; Baranwal et al., 2019; Jia et al., 2020; Liang et al., 2020; Liu H. et al., 2020; Zhou et al., 2020a,b; Zhu et al., 2021). For convenience, these algorithms were performed with their default parameters, which are set in the corresponding platform.

#### RF

It is an assembly classification algorithm that contains several DTs. Each DT is built by randomly selecting samples and features from the original dataset. For a query sample, each DT provides the prediction class. RF integrates these prediction classes with majority voting, i.e., the class receiving most votes is the predicted class of RF. Although DT is a relatively weak classification algorithm, RF is much stronger. The current study adopted the Scikit-learn package to implement RF.

#### SVM

It can transform data with a nonlinear pattern from original low-dimensional data space to a new high-dimensional data space, where the data display a linear pattern. Then, it divides the data points in such high-dimensional space, requiring data-interval maximization among different data classes/groups. It could predict the class or group of a new sample by determining the interval to which this new sample data belongs. Here, the tool “SMO” in Weka was adopted to construct SVM classifiers. The training procedure of this SVM is optimized by the sequential minimal optimization algorithm (Platt, 1998).

#### kNN

It is one of the most classic classification algorithms. For a test sample, it initially computes the distance between it and the training samples. Then, it ranks all training samples with the increasing order of the distances. Next, it selects the *k* high-ranked training samples (i.e., nearest k neighbors) and further estimates the label distribution of these *k* samples. The label distribution is then used to help predict the class of test sample, i.e., the class label with the highest frequency in the label distribution. The tool “IBk” in Weka was performed for kNN classifier building.

#### DT

Different from the above three classification algorithms, which can only be used to construct black-box classifiers, DT can construct human understanding classification and regression models by using interpretative rules. Generally, it indicates individual features’ roles and weights in classification or regression models by using the IF–TEHN format. Here, the CART algorithm with the Gini index in the Scikit-learn package was used for DT classifier construction.

## Results and Discussion

**FIGURE 1:**
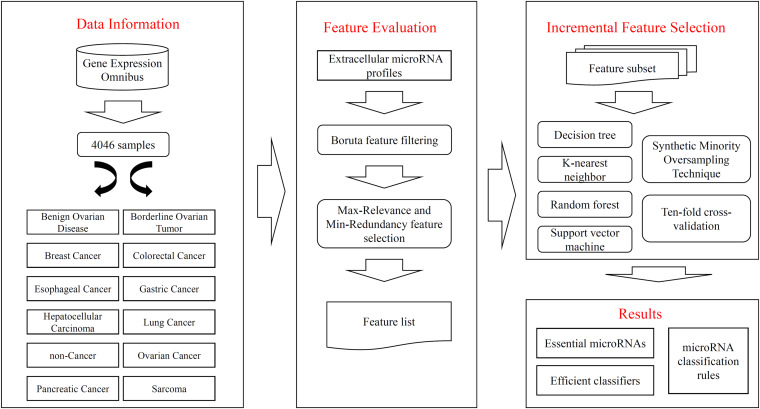
Flow chart to show entire procedures. The original data are retrieved from Gene Expression Omnibus, which contains 4046 samples and 12 classes. The extracellular microRNA profiles are analyzed by Boruta and mRMR methods one by one, resulting in a feature list. The list is fed into the IFS method, incorporating four classification algorithms, SMOTE and ten-fold cross-validation, to extract essential microRNAs, construct efficient classifiers, and set up classification rules.

In this study, we gave a computational investigation on the extracellular microRNA dataset of multiple cancer types. Some feature selection methods and classification algorithms were adopted. The entire procedures are illustrated in Figure 1. This section first introduced the results and then gave an extensive discussion.

### Results of Boruta and mRMR Methods

We first applied the Boruta method to the extracellular microRNA dataset for discarding non-essential features (microRNAs). As a result, 1849 features were excluded and 716 features were kept. These remaining features are provided in Supplementary Table S1.

For the remaining 716 features, they were further analyzed by the mRMR method. As mentioned in Section “Max-Relevance and Min-Redundancy Feature Selection”, a feature list, mRMR feature list, was generated, in which features were ranked according to their importance. This list is also provided in Supplementary Table S1.

### Results of IFS Method With Different Classification Algorithms

**FIGURE 2:**
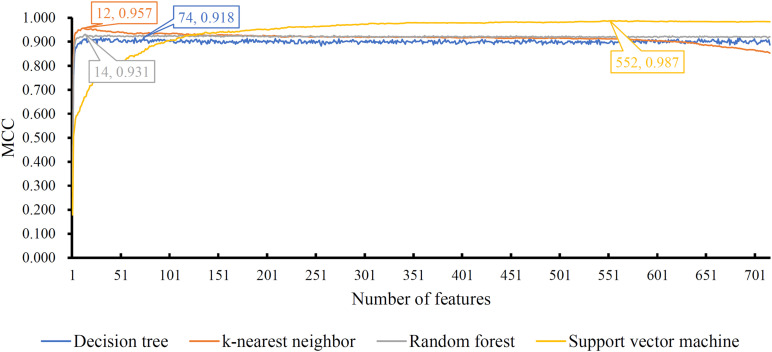
IFS curves with different classification algorithms on different number of features. Four algorithms yielded the highest MCCs with top 74, 12, 14, and 552 features.

**FIGURE 3:**
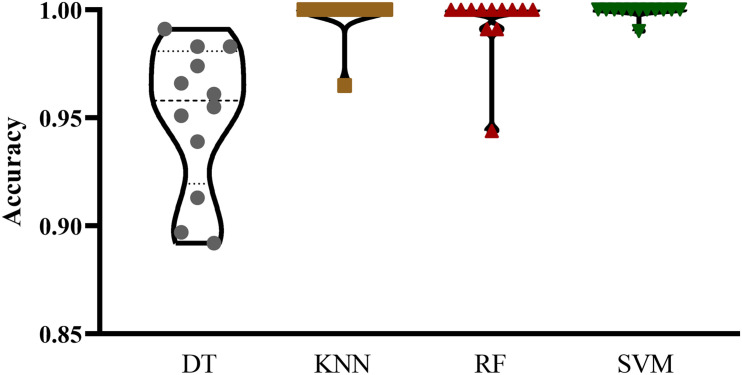
Violin plot to show accuracies on 12 classes yielded by the optimum classifiers with four different classification algorithms. The optimum SVM classifier was best.

**FIGURE 4:**
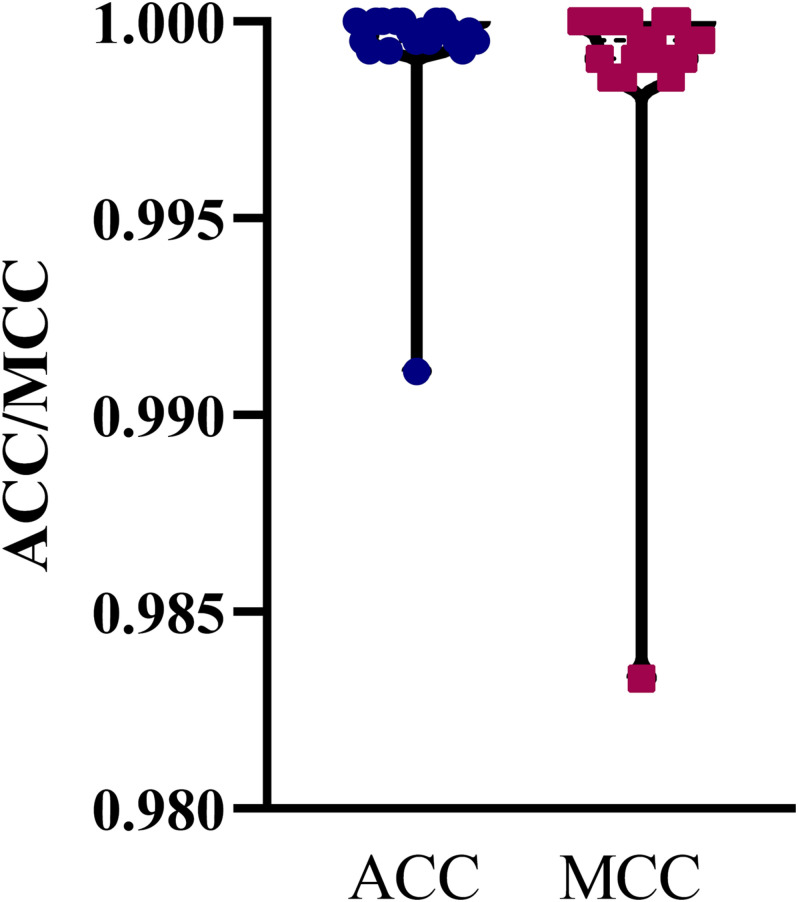
Violin plot to show ACCs and MCCs yielded by the optimum SVM classifier under 10-fold cross-validation 20 times. ACC and MCC vary in a small interval, suggesting the stability of the optimum SVM classifier.

The mRMR feature list generated by mRMR method was fed into the IFS method. Using an interval step of 1, many feature subsets were extracted, e.g., the first feature subset contained the top-ranked feature, and the second feature subset contained the two top-ranked features. For each feature subset and one of the four classification algorithms (SVM, RF, kNN, and DT), a classifier was built on samples represented by features in the subset. Ten-fold cross-validation (Kohavi, 1995) was adopted to evaluate the performance of each classifier. Notably, SMOTE was applied when assessing the performance of each classifier. Results were counted as the following measurements: accuracy on each class, overall accuracy (ACC) and Matthew correlation coefficient (MCC) (Matthews, 1975; Gorodkin, 2004). These measurements are available in Supplementary Table S2. For an easy observation, one IFS curve was plotted for each classification algorithm, in which MCC was set as the Y-axis and number of used features was set as the X-axis, which is shown in Figure 2. For kNN, the highest MCC was 0.957 when top 12 features were used. Accordingly, the optimum kNN classifier was built using these 12 features. The highest MCC of RF was 0.931, which was obtained by the top 14 features. The optimum RF classifier with these top 14 features can be set up. As for SVM, the highest MCC was 0.987 when top 552 features were adopted. It was higher than that of the optimum kNN or RF classifiers. The ACCs of above three optimum classifiers are listed in Table 2. The ACC of the optimum SVM classifier was also highest. The accuracies on 12 classes yielded by these optimum classifiers are illustrated in Figure 3. Evidently, the optimum SVM classifier was also best. Because the partition of the 10-fold cross-validation can influence the evaluation results, we further tested the performance of the optimum SVM classifier with ten-fold cross-validation 20 times. Obtained ACCs and MCCs are illustrated in Figure 4. The ACCs varied between 0.990 and 1.000, whereas MCCs were between 0.980 and 1.000, indicating that such optimum classifier was quite stable and above results can be believable.

**TABLE 2:** Performance of IFS with four different classification algorithms.

Classification algorithm	Number of features	ACC	MCC
Decision tree	74	0.955	0.918
k-nearest neighbor	12	0.976	0.957
Random forest	14	0.961	0.931
Support vector machine	552	0.993	0.987

**FIGURE 5:**
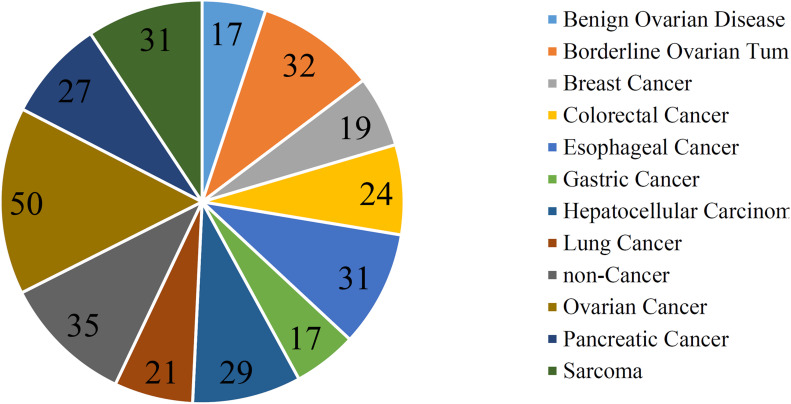
Pie chart to show the number of rules for each class.

In addition to three black-box classification algorithms, we also employed a white-box algorithm, DT, to do the same test. The IFS results are also provided in Supplementary Table S2 and the IFS curve was plotted in Figure 2. The optimum DT classifier produced the MCC of 0.918, which was based on the top 74 features. The corresponding ACC was 0.955, which is listed in Table 2. The ACC and MCC were lower than those of the above-mentioned three optimum classifiers. Furthermore, the accuracies on 12 classes of the optimum DT classifier are shown in Figure 3. They were also lower than those of other three optimum classifiers. Although the performance of the optimum DT classifier was lower than other three optimum classifiers, it can provide a clear classification procedure, thereby providing more insights to investigate different cancer types. In view of this, we constructed a DT based on the top 74 features, which were used to build the optimum DT classifier. Then, 333 microRNA rules were extracted from such DT, which are available in Supplementary Table S3. Each class was assigned to some rules, where the number of rules (50) on “ovarian cancer” was most, followed by “non-cancer.” The numbers of rules on “benign ovarian disease” and “gastric cancer” were least, which were only 17. The number of rules for each class is shown in Figure 5.

Here, a group of qualitative microRNAs (features) and quantitative microRNA rules were identified to contribute to detailed cancer-classification recognition. According to recent publications, the top-ranked optimal features and rules were supported and validated with the respective cancer-subtype specific pathological roles, which will be discussed in Sections “Optimal MicroRNAs Contributing to Cancer Classification” and “Optimal MicroRNA Rules Contributing to Cancer Classification”.

### Optimal MicroRNAs Contributing to Cancer Classification

By analyzing the shared extracellular microRNA dataset, we identified a group of microRNAs that can effectively distinguish different cancer subtypes but not cancer or controls, reflecting the internal differences among different cancer subtypes. This section selected the top 10 microRNAs in the mRMR feature list for detailed analysis, which are listed in Table 3.

**TABLE 3:** Top 10 microRNAs identified by Boruta and mRMR methods.

Rank	miRbase accession number	microRNA (Full name)
1	MIMAT0022259	hsa-miR-5100
2	MIMAT0023713	miR-6088
3	MIMAT0019071	miR-4532
4	MIMAT0027392	miR-6746
5	MIMAT0031000	miR-8073
6	MIMAT0027500	miR-6800
7	MIMAT0019776	miR-1343
8	MIMAT0019947	miR-4783
9	MIMAT0000278	miR-221
10	MIMAT0019957	miR-4787

The first identified microRNA was hsa-miR-5100 (MIMAT0022259). According to recent publications, this microRNA has been identified in multiple tumor-related studies and is functionally correlated with tumorigenesis (Tang et al., 2014; Wang et al., 2016; Jacob et al., 2018; Tian et al., 2020). However, it has been confirmed to have a specific expression level only in plasma in colon cancer (Jacob et al., 2018) and in extracellular matrix in oral carcinoma (Kawakubo-Yasukochi et al., 2018). Accordingly, predicting this microRNA to have discriminative capacity in 11 candidate cancer subtypes is reasonable.

The next predicted microRNA signature was miR-6088 (MIMAT0023713). It has also been identified in only three cancer subtypes, namely nasopharyngeal cancer (Li K. et al., 2020), ovarian cancer (Pandey et al., 2019), and melanoma (Wozniak et al., 2017), thereby confirming its classification capacity for ovarian cancer in our dataset. The third predicted signature, miR-4532 (MIMAT0019071), has also been regarded as a potential circulating extracellular cancer biomarker according to previous studies (Fiorino et al., 2016; Pascut et al., 2019; Zhao et al., 2019), including hepatocellular carcinoma (Fiorino et al., 2016) and leukemia (Zhao et al., 2019).

As regards the two microRNAs miR-6746 (MIMAT0027392) and miR-8073 (MIMAT0031000), both reportedly participate in specific cancer-associated tumorigenesis, corresponding with our prediction. For miR-6746, it has been shown to have specific expression level in the plasma of pancreatic cancer patients but not in those of other patients (Sheng et al., 2020). For miR-8073, it has been identified in both pancreatic (Shams et al., 2020) and breast (Cui et al., 2018) cancers, implying that such microRNA may distinguish two cancer subtypes from the other cancer subtypes and normal controls.

The next microRNA, miR-6800 (MIMAT0027500), is also reportedly a potential biomarker for prostate (Liu H.P. et al., 2020) and colorectal (Yan et al., 2017) cancers, confirming its capacity for distinguishing colorectal cancer from 11 other cancer subtypes and normal controls in this analysis.

The remaining microRNAs, namely miR-1343 (MIMAT0019776), miR-4783 (MIMAT0019947), miR-221 (MIMAT0000278), and miR-4787 (MIMAT0019957), have also been confirmed to contribute to specific cancer subtypes [e.g., lung adenocarcinoma correlated with miR-1343 (Zhang X. et al., 2020), rectal cancer correlated with miR-4783 (Mullany et al., 2016), prostate cancer correlated with miR-221 (Agaoglu et al., 2011), and pancreatic cancer correlated with miR-4787 (Mody et al., 2016)], thereby further validating the efficacy and accuracy of our newly established computational workflow.

### Optimal MicroRNA Rules Contributing to Cancer Classification

In addition to the above identified microRNA si+oldd gnatures, we recognized and established a series of quantitative classification rules for more interpretable cancer classification. Due to the limitation of the manuscript’s length, we selected one representative rule for each specific cancer classification for subsequent detailed discussions, including 11 cancer subtypes and 1 normal control.

The first rule for the identification of Benign Ovarian Disease is rule 58, involving 14 different microRNAs. Among these microRNAs, a specific microRNA named as miR-5100 (MIMAT0022259) has been detected in the plasma of benign ovarian cysts, which can be classified into benign ovarian disease, corresponding with our prediction (Zhang L. et al., 2020). As for Borderline Ovarian Tumor, rule 72 has been confirmed to contribute to the identification of patients with such disease. Among multiple microRNA biomarkers, the significant one is also miR-5100 (MIMAT0022259), indicating that it is still an ovarian-associated signature. Moreover, miR-296 (MIMAT0000690) has been predicted to be correlated with Borderline Ovarian Tumor, whose correlation has also been verified (Li Y. et al., 2020). For breast cancer, as discussed above, miR-8073 (MIMAT0031000) shown in rule 145 has been validated to be related to breast cancer with relatively high expression level (Cui et al., 2018). Similarly, miR-6800 (MIMAT0027500) of colorectal cancer shown in rule 274 has been discussed above (Yan et al., 2017), indicating a relatively low expression level of such microRNA compared with normal controls and other cancer subtypes.

For esophageal cancer and gastric cancer, the optimal quantitative microRNA features in the rules have also been validated. In esophageal cancer, as described in rule 13, miR-6784 (MIMAT0027468) has been shown to have a relatively high expression level and validated by recent publications (Fujihara et al., 2015). As for gastric cancer-associated signatures at the microRNA level, miR-3663 (MIMAT0018085) has been shown to be a potential biomarker for gastrointestinal tumors, including gastric cancer (Lee et al., 2016; Xu et al., 2018; Kubo et al., 2019). To specifically identify gastric cancer, another microRNA named miR-1343 (MIMAT0019776) has been shown to be a specific gastric cancer-associated microRNA by regulating TEAD4 (Zhou et al., 2017), thereby validating our prediction.

As regards class hepatocellular carcinoma, lung cancer, and ovarian cancer, we also identified specific classification rules with the specific microRNA signatures discussed above. For hepatocellular carcinoma, miR-4532 (MIMAT0019071) has been shown to be a decisive biomarker with a relatively low expression level (Fiorino et al., 2016) in rule 158, corresponding with our discussion above. In lung cancer-associated rules, a typical rule named rule 162 has been shown to have a relatively high expression level of miR-1343 (MIMAT0019776) in patients’ plasma compared with normal controls and other patients with other cancer subtypes (Zhang X. et al., 2020). Similar rules have been established for ovarian cancer involving miR-6088 (Pandey et al., 2019), implying the reliability of our predicted rules.

For pancreatic cancer and sarcoma, miR-6746 (MIMAT0027392) shown as a significant parameter in rule 207 has also been confirmed to be correlated with and be specific for pancreatic cancer, as discussed above (Sheng et al., 2020), confirming the efficacy of our prediction. For sarcoma, miR-92B (MIMAT0004792) shown in rule 9 has been presented to be up-regulated in sarcoma compared with other cancer subtypes and no cancer controls. According to recent publications, in 2017, researchers already confirmed that miR-92B is a novel biomarker for carcinoma monitoring (Uotani et al., 2017), corresponding with our prediction. Apart from the discussion above, individuals with extracellular microRNA profiling not satisfying either of the above rules may be classified into controls.

## Conclusion

As discussed above, our identified optimal microRNA signatures and related quantitative classification rules have all been verified by recent publications, helping us classify different cancer subgroups and non-cancer controls. For the first time, we integrated feature selection and machine-learning models with inherited information at the extracellular microRNA level to present a new workflow for cancer-classification recognition, early diagnosis, and monitoring with high prediction specificity. The promising results obtained in this study (microRNA signatures and rules) may validate the specific and diverse roles of extracellular microRNAs during tumorigenesis and may also lay a solid foundation for further studies on the potentials of extracellular microRNAs on tumor diagnosis and monitoring.

## Data Availability Statement

Publicly available datasets were analyzed in this study. This data can be found here: https://www.ncbi.nlm.nih.gov/geo/query/acc.cgi?acc=GSE106817.

## Ethics Statement

Written informed consent was not obtained from the individual(s) for the publication of any potentially identifiable images or data included in this article.

## Author Contributions

TH and Y-DC designed the study. FY, LC, TZ, and SD performed the experiments. FY, ZL, TZ, and Y-HZ analyzed the results. FY, ZL, LC, and TZ wrote the manuscript. All authors contributed to the research and reviewed the manuscript.

## Conflict of Interest

The authors declare that the research was conducted in the absence of any commercial or financial relationships that could be construed as a potential conflict of interest.

## Footnotes

1 https://www.ncbi.nlm.nih.gov/geo/query/acc.cgi?acc=GSE106817

2 https://www.cs.waikato.ac.nz/ml/weka

## Supplementary Material

Supplementary Table 1 Features filtered by Boruta and their ranks generated by mRMR.

Supplementary Table 2 Performance of IFS with different classifiers.

Supplementary Table 3 Classification rules generated by decision tree.
